# Do Political and Economic Choices Rely on Common Neural Substrates? A Systematic Review of the Emerging Neuropolitics Literature

**DOI:** 10.3389/fpsyg.2016.00264

**Published:** 2016-02-25

**Authors:** Sekoul Krastev, Joseph T. McGuire, Denver McNeney, Joseph W. Kable, Dietlind Stolle, Elisabeth Gidengil, Lesley K. Fellows

**Affiliations:** ^1^Department of Neurology and Neurosurgery, Montreal Neurological Institute, McGill UniversityMontreal, QC, Canada; ^2^Center for Cognitive Neuroscience, Department of Psychology, University of PennsylvaniaPhiladelphia, PA, USA; ^3^Centre for the Study of Democratic Citizenship, Department of Political Science, McGill UniversityMontreal, QC, Canada

**Keywords:** neuroeconomics, decision-making, reward, heuristics, functional MRI, meta-analysis

## Abstract

The methods of cognitive neuroscience are beginning to be applied to the study of political behavior. The neural substrates of value-based decision-making have been extensively examined in economic contexts; this might provide a powerful starting point for understanding political decision-making. Here, we asked to what extent the neuropolitics literature to date has used conceptual frameworks and experimental designs that make contact with the reward-related approaches that have dominated decision neuroscience. We then asked whether the studies of political behavior that can be considered in this light implicate the brain regions that have been associated with subjective value related to “economic” reward. We performed a systematic literature review to identify papers addressing the neural substrates of political behavior and extracted the fMRI studies reporting behavioral measures of subjective value as defined in decision neuroscience studies of reward. A minority of neuropolitics studies met these criteria and relatively few brain activation foci from these studies overlapped with regions where activity has been related to subjective value. These findings show modest influence of reward-focused decision neuroscience on neuropolitics research to date. Whether the neural substrates of subjective value identified in economic choice paradigms generalize to political choice thus remains an open question. We argue that systematically addressing the commonalities and differences in these two classes of value-based choice will be important in developing a more comprehensive model of the brain basis of human decision-making.

## Introduction

Political choice matters. Whether expressed in the voting booth or in response to a pollster, individual choices collectively influence the direction of public policy and ultimately the well-being of society and the individuals that compose it (Soroka and Wlezien, 2010). Cognitive neuroscience offers novel methods for understanding the mechanisms underlying decision-making, but political scientists have been slower than economists to apply these methods. This has been variously attributed to an excessive preoccupation with external validity (Albertson and Brehm, 2003), the “grip of environmental determinism,” political correctness, and the belief that politics is somehow *sui generis* and uniquely human (Hibbing and Smith, 2007; Alford and Hibbing, 2008). There are also practical reasons: political concepts may not lend themselves as readily as economic concepts to investigation with cognitive neuroscience methods (Tingley, 2006). However, political scientists have begun to recognize that neuroscientists and political scientists are not necessarily “strange bedfellows” (Cacioppo and Visser, 2003) and are turning to cognitive neuroscience to gain a deeper understanding of political behavior (Fowler and Schreiber, 2008).

In economic contexts, it is assumed that choices are made to maximize subjective value. The neural basis of value-related processes has thus been the focus of much of the neuroeconomics work to date, yielding insights into the mechanisms underlying economic behavior (Fellows, 2004; Rangel et al., 2008; Kable and Glimcher, 2009; Padoa-Schioppa, 2011; Rangel and Clithero, 2013). There is also evidence that these mechanisms may be involved in value-based choice more generally, beyond strictly economic contexts. For example, tasks requiring value assessment of social or esthetic stimuli have reported value-related signals similar to those observed in economic paradigms (Lin et al., 2012; Bartra et al., 2013; Ruff and Fehr, 2014; Pegors et al., 2015). Here, we ask if the neural substrates identified for economic behaviors are also engaged in political decision-making contexts. A “yes” to this question could accelerate neuropolitics research, capitalizing on the advances in economic domains. A “no” would be equally valuable, clarifying the research priorities for both areas of study to achieve a more complete understanding of the neuroscience of human decision-making.

Current neuroeconomic models propose that the assignment of value to alternatives likely involves a set of variables that represent internal states (e.g., hunger) and external factors (e.g., risk, delay) relevant to the consequences of each option. There is converging evidence that the orbitofrontal cortex (OFC), ventromedial prefrontal cortex (vmPFC), and associated ventral striatum carry information related to the subjective value of choice alternatives. Functional neuroimaging studies in humans have shown value-related signals in vmPFC and ventral striatum in a wide range of paradigms, involving many types of reward, such as food, money, and social reward such as attractive faces (for reviews, see Bartra et al., 2013; Ruff and Fehr, 2014). Ventromedial frontal lesions in humans disrupt value-based preference judgments across several stimulus types (Fellows and Farah, 2007; Moretti et al., 2009; Henri-Bhargava et al., 2012), and electrophysiological studies in monkeys show that activity of neurons in the OFC reflect changes in stimulus value (reviewed in Padoa-Schioppa, 2011). This literature supports the idea that there are common neural mechanisms encoding value-related information across a range of contexts.

Political science has also used models based on value-maximization concepts to understand political choices, providing a conceptual connection to economics-oriented decision neuroscience. Indeed, one of the classic contributions to the voting behavior literature was entitled, An Economic Theory of Democracy (Downs, 1957). Downs’ model assumes that voters will act deliberately by choosing the party that maximizes their value-gain, suggesting that political choice involves the same decision-making calculus as economic choice.

However, political scientists have traditionally been more skeptical than economists of utility maximizing models (Green and Shapiro, 1994; Jones, 1999). One example of the failure of these models pertains to the decision to vote or not. According to the “rational choice model,” a person will vote if the expected benefit of voting exceeds the cost, but for the vast majority of voters the expected benefit is virtually nil because the chances of casting the decisive vote are trivially small (Blais, 2001). The fact that many people nonetheless do turn out to vote has been called the “paradox that ate rational choice theory” (Fiorina, 1981). Still, the rational choice model remains a prominent approach to understanding political decision-making. Neuroscience models of value-based decision-making based on economic perspectives may thus be relevant for understanding the brain mechanisms supporting political choice.

Here, we address this question empirically, first asking to what extent the concept of subjective value has been applied to study the neural substrates of political choice, and second asking whether that literature provides evidence of common neural mechanisms underlying value-based economic and political choices. We systematically reviewed the neuropolitics literature, asking whether existing studies in this emerging field used designs that were sufficiently similar to the literature on reward-related subjective value to allow comparison. The majority of the studies so identified used the same method, functional MRI (fMRI), allowing the results to be summarized quantitatively in relation to regions commonly associated with subjective value signaling in economic or other reward-related decision paradigms. We compared the results of these studies to the findings from a recent meta-analysis of fMRI studies of value-based choice, mainly assessed with economic paradigms, to assess whether there are common regional activation patterns related to subjective value across these two literatures.

## Materials and Methods

### Literature Search

A systematic literature search was conducted in May 2014 to identify papers discussing the neural basis of political behavior in humans. The search terms “politics,” “political,” “Democrat,” “Republican,” “brain” and “neuroscience” were used on Google Scholar and Web of Knowledge. The database searches were supplemented by manual review of the citations in these papers. Papers were included in the first stage of the literature review if their central focus was the link between political behavior and the brain, whether review articles or experimental studies. This yielded 27 papers investigating topics such as face judgment in political contexts, partisanship, motivated reasoning, political interest, political attitudes and automatic processing of political preference.

In a second step, we applied the inclusion and exclusion criteria that were used in a recent meta-analysis of over 200 fMRI studies investigating value-based choice (Bartra et al., 2013) to this body of neuropolitics research. The reference meta-analysis included studies containing the keywords “fMRI” and “reward” and identified brain regions where activity was consistently related to behavioral measures of positive and negative subjective value across a wide variety of value-based decision-making tasks. Subjective value, conceived of as the “common currency” value attributed to available alternatives, was either directly measured through ratings or preference judgments or, in the case of monetary reward, inferred *a priori* as being higher for larger amounts of money. None of the neuropolitics papers identified in the present search were captured by that meta-analysis.

The same criteria applied to our sample of neuropolitics literature yielded English-language papers in which BOLD signal was measured with fMRI, as a function of positive and/or negative subjective value. As in Bartra et al. (2013), we did not limit ourselves to studies that used particular tasks or stimuli, instead accepting any experimental design that yielded a clear behavioral measure of subjective value, such as voting for a candidate based on a photograph of his/her face (positive subjective value) or rating a policy negatively on a visual analog scale (negative subjective value). Only experiments that used whole brain analyses to report peak activation foci in stereotactic spatial coordinates (Talairach or MNI space) and linked those activations to either positive or negative subjective value measures were included.

For the studies meeting these criteria, Talairach coordinates were converted to MNI space and activation foci were coded according to whether they corresponded to positive or negative subjective value. The list of coordinates so identified are those voxels that showed significantly increased BOLD signal in relation to a behavioral measure of either higher or lower subjective value in a political task. Although the sample size was insufficient to provide a full formal test of whether there were consistent patterns across the political studies, we nonetheless followed the same methodology to provide at least a qualitative sense of common patterns of activation. First, we looked for common patterns of activation related to positive or negative subjective value across the neuropolitics studies, tested against the null hypothesis that foci were distributed randomly, taking into account the probability of gray matter in each voxel (derived from the ICBM Tissue Probabilistic Atlases; http://www.loni.usc.edu/ICBM/Downloads/Downloads_ICBMprobabilistic.shtml), as in Bartra et al. (2013).

Next, we asked whether any activation foci from the neuropolitics studies fell within the regions previously identified as consistently relating to subjective value in economic paradigms (Bartra et al., 2013). A region-of-interest (ROI) was established for positive and negative subjective value based on the published meta-analysis. Two masks (one each for positive and negative subjective value) were then generated in FSL (Smith et al., 2004). The coordinates that remained after applying these masks represented the overlap between foci associated with subjective value in the politics studies and the regions consistently related to subjective value in the reference meta-analysis. For visualization purposes, a 5 mm radius sphere was centered on each coordinate passed through the masking phase. Finally, given that laterality was not tested in the source studies for political subjective value, this step was repeated, collapsing across hemispheres, in an exploratory effort to maximize our ability to detect common activation patterns in this small sample.

## Results

The initial literature search, performed in May 2014, yielded 27 papers (**Table 1**) that corresponded to our search terms and inclusion criteria. Of these, 10 were review papers, and 17 reported primary data. In the latter set, six focused on political attitudes and emotion, three on party identification, two on political interest, and nine on specific processes carried out in political judgment contexts (face judgment, automatic processing and motivated reasoning). Clearly, neuropolitics is still an emerging field with relatively few original research reports to date (Fowler and Schreiber, 2008).

**Table 1:** Summary of the neuropolitics literature identified by the systematic search strategy, classified by the primary method used, with the studies that met inclusion criteria for the fMRI meta-analysis highlighted in bold.

Reference	Title	Topic	Method
Jost and Amodio, 2012	Political ideology as motivated social cognition: behavioral and neuroscientific evidence	Partisanship – Motivated reasoning	Structural MRI
Kanai et al., 2011	Political orientations are correlated with brain structure in young adults	Partisanship – Party identification	Structural MRI
Oxley et al., 2008	Political attitudes vary with physiological traits	Attitude	Psychophysiology
Dawes and Fowler, 2009	Partisanship, voting, and the dopamine D2 receptor gene	Partisanship – Interest	Genetics
Cunningham et al., 2004	Implicit and explicit evaluation: fMRI correlates of valence, emotional intensity, and control in the processing of attitudes	Automatic processing	fMRI
**Gozzi et al., 2010**	**Interest in politics modulates neural activity in the amygdala and ventral striatum**	**Partisanship - Interest**	**fMRI**
**Kaplan et al., 2007**	**Us versus Them: political attitudes and party affiliation influence neural response to faces of presidential candidates**	**Face judgment**	**fMRI**
**Kato et al., 2009**	**Neural correlates of attitude change following positive and negative advertisements**	**Attitude**	**fMRI**
Knutson et al., 2006	Politics on the brain: an fMRI investigation	Face judgment	fMRI
**Rule et al., 2010**	**Voting behavior is reflected in amygdala response across cultures**	**Face judgment**	**fMRI**
Schreiber et al., 2013	Red brain, blue brain: evaluative processes differ in democrats and republicans	Partisanship – Party identification	fMRI
**Spezio et al., 2008**	**A neural basis for the effect of candidate appearance on election outcomes**	**Face judgment**	**fMRI**
**Tusche et al., 2013**	**Automatic processing of political preferences in the human brain**	**Automatic processing**	**fMRI**
**Westen et al., 2006**	**Neural bases of motivated reasoning: an fMRI study of emotional constraints on partisan political judgment in the 2004 U.S. presidential election**	**Partisanship - Motivated Reasoning**	**fMRI**
Zamboni et al., 2009	Individualism, conservatism, and radicalism as criteria for processing political beliefs: a parametric fMRI study	Attitude	fMRI
Amodio et al., 2007	Neurocognitive correlates of liberalism and conservatism	Partisanship – Party identification	EEG
Dhont et al., 2011	A step into the Anarchist’s mind: examining political attitudes and ideology through event-related Brain Potentials	Attitude	EEG
Fowler and Schreiber, 2008	Biology, politics, and the emerging science of human nature	Review	Commentary
Friend and Thayer, 2011	Brain imaging and political behavior: a survey	Review	Commentary
Lieberman et al., 2003	Is political cognition like riding a bicycle? how cognitive neuroscience can inform research on political thinking	Review	Commentary
Marcus, 2000	Emotions in politics	Review	Commentary
Marcus et al., 1998	Linking neuroscience to political intolerance and political judgment	Review	Commentary
McDermott, 2009	The case for increasing dialog between political science and neuroscience	Review	Commentary
Schreiber, 2004	Political cognition as social cognition: are we all political sophisticates?	Review	Commentary
Spezio and Adolphs, 2007	Emotional processing and political judgment: toward integrating political psychology and decision neuroscience	Review	Commentary
Theodoridis and Nelson, 2012	Of BOLD claims and excessive fears: a call for caution and patience regarding political neuroscience	Review	Commentary
Tingley, 2006	Neurological imaging as evidence in political science: a review, critique, and guiding science	Review	Commentary

Seven of these 17 original studies met the inclusion and exclusion criteria applied in the Bartra et al. (2013) meta-analysis (**Table 2**). The 10 studies reporting primary data that did not meet inclusion criteria examined neurobiological correlates of individual differences in political traits (i.e., specific party affiliation, liberalism, etc.) rather than studying evaluative behaviors or choices that could yield a contrast related to subjective value. Thus, just under half of the existing neuropolitics studies (7/17) provided primary data relating subjective value in political contexts to the brain.

**Table 2:** Neuropolitics studies reporting BOLD signal in relation to behavioral measures of positive or negative subjective value.

Subjective value valence	Topic	Task	Reference	Sample size	Sample characteristics	Country	Regions with activation overlapping with economic meta-analysis (MNI coordinates)
Positive	Partisanship and political interest	Agree versus disagree with political opinions in interested versus uninterested subjects	Gozzi et al., 2010	25	Non-Partisan	USA	Putamen (24,15,-3), (24,-3,-18)
	Automatic processing of faces	*Post-hoc* ratings of politicians that are shown while subject is engaged in a distractor task	Tusche et al., 2013	20	Non-Partisan	Germany	Medial Temporal Lobe, Caudate (-6,14,4)
	Face judgment	Voting for a political candidate	Rule et al., 2010	28	Non-Partisans	USA, Japan	None
	Political attitude	Changed preference for politician after Positive Political Ad	Kato et al., 2009	40	Partisans	USA	None
Negative	Face judgment	Democrats and Republicans viewing candidate from opposing party versus one from their own	Kaplan et al., 2007	20	Partisans	USA	Insula (-6,14,2)
	Face judgment	Not voting for a political candidate	Spezio et al., 2008	24	Non-Partisans	USA	Insula, Dorsal Anterior Cingulate, Thalamus (45,9,-3), (-3,-27,-3), (-9,21,24)
	Partisanship – motivated reasoning	Viewing information threatening to a political candidate from subject’s own party versus neutral	Westen et al., 2006	30	Partisans	USA	None
	Political attitude	Changed preference for politician after negative political Ad	Kato et al., 2009	40	Partisans	USA	None

The seven studies that met inclusion criteria reported data from a total of 187 subjects. Three of these seven studies investigated face judgment in political contexts, while the remaining four studied motivated reasoning, political interest, attitude change in response to advertising and automatic processing of political preference. Across these 7 fMRI studies, reporting either a binary contrast or a continuous parametric analysis in a total of 13 tasks, four studies reported activations linked to behavioral measures of positive subjective value (for example, positively rating a politician) and four reported activations related to behavioral measures of negative subjective value (for example, negatively rating a politician) (**Table 2**).

**FIGURE 1:**
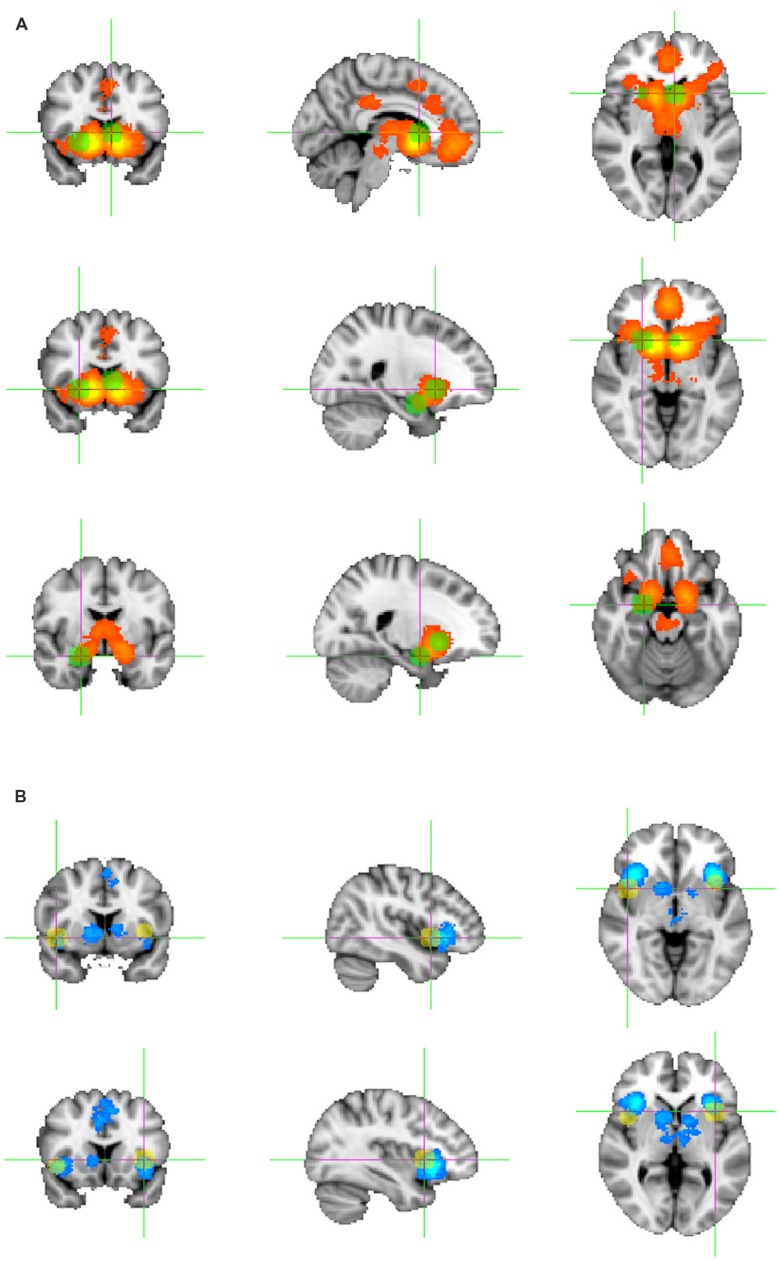
**fMRI foci related to subjective value in the neuropolitics studies that overlapped with the subjective value-associated ROI’s extracted from a large-scale meta-analysis of studies of reward processing using a range of (largely economic) paradigms (Bartra et al., 2013), shown on the standard MNI brain. (A)** Foci (in green) overlapping with the ROI for positive subjective value. **(B)** Foci (in yellow) overlapping with the ROI for negative subjective value. MNI coordinates for these foci are provided in **Table 2**.

The whole-brain analysis of above chance clustering of activation foci across the four political studies reporting activations associated with positive subjective value and the four political studies reporting activations associated with negative subjective value did not yield any overlap greater than would be expected by chance, even when collapsed across hemispheres. The maximum overlap at a single location (5 mm radius around a reported coordinate) was 75% (i.e., common activation focus in three of four studies) for contrasts related to positive subjective value and 50% (i.e., common activation foci identified in two of four studies) for contrasts related to negative subjective value. With this sample size, foci would need to overlap in all four studies to be distinguishable from chance. **Figure 1** shows the foci of activation in both hemispheres related to subjective value in political contexts overlaid on the ROIs identified in Bartra et al. (2013).

Perhaps the most important finding of this systematic review is that only a handful of fMRI studies of neuropolitics met criteria for inclusion, whereas the same criteria yielded 206 studies reporting on subjective value in other (principally economic) decision neuroscience paradigms. Few foci related to subjective value in the politics studies fell within areas that the prior meta-analysis identified as commonly carrying signals related to either positive or negative subjective value in economic or other reward contexts. Of all the foci reported in the political studies (130 coordinates in total), only five passed the masking stage of our ROI analysis (**Figure 1**), i.e., fell within the brain regions consistently related to subjective value in reward-based decision making paradigms, a number not different from chance in this small sample. An additional two foci associated with negative subjective value were identified when the analysis was repeated, collapsed across hemispheres (**Table 2**).

Keeping the preliminary nature of these observations in mind, we briefly summarize the neuropolitics studies that showed overlap with the existing reward-related decision literature, as a starting point for future work. The foci associated with positive subjective value were from studies by Gozzi et al. (2010) and Tusche et al. (2013). Gozzi et al. (2010) used a task that measured agreement with political opinions in groups of subjects varying in level of political interest. Agreement with political opinions in politically interested versus uninterested subjects was considered as a measure of positive subjective value, and was related to activation in the putamen. In Tusche et al. (2013), subjects were shown photographs of familiar politicians while engaged in a distractor task. Positive ratings of these politicians were related to activation in the medial temporal lobe and caudate.

The foci associated with negative subjective value were reported in studies by Kaplan et al. (2007) and Spezio et al. (2008). Kaplan et al. (2007) studied partisans viewing photographs of politicians of an opposing party versus their own party. Spezio et al. (2008) asked subjects to vote for unfamiliar politicians based on head and shoulders photographs. Both studies found BOLD activation in the insula that was related to viewing a political candidate that the subject disliked. The coordinates in these two studies did not overlap, however. Two additional foci from the Spezio et al. (2008) study, in the dorsal anterior cingulate and thalamus, were identified when laterality was not considered.

## Discussion

Neuroscience research on economic decision-making now constitutes a relatively large body of work, yielding detailed models of value-based decision-making (Fellows, 2004; Kable and Glimcher, 2009; Padoa-Schioppa, 2011; Rangel and Clithero, 2013). Much of this work has used fMRI, and found consistent activation related to subjective value in several brain regions in a range of reward-related tasks (reviewed in Bartra et al., 2013), arguing that these regions support domain-general value-related processes. There is also some converging evidence from human lesion studies that damage to the ventral frontal lobe, including vmPFC and OFC, impairs value-based choices across a range of contexts, including political choices, supporting the claim that this region is necessary for value-based decisions, broadly defined (Fellows and Farah, 2007; Camille et al., 2011; Henri-Bhargava et al., 2012; Xia et al., 2015).

In this systematic review, we found that there are relatively few neuropolitics fMRI studies that have made use of designs or analytic frameworks directly comparable to neuroeconomic studies. Although the handful of studies that did meet our criteria does not support a robust quantitative analysis, it is notable that even at a qualitative level there were no consistent foci associated with subjective value in political contexts. Although overlapping patterns of activation are not a definitive indication that a brain region is similarly engaged by two tasks (Leech et al., 2012; Woo et al., 2014), this is nonetheless a reasonable starting point for testing the generality of the mapping of value-related processes to the brain across economic and political contexts. The absence of consistent patterns here could suggest that the neuroeconomics framework, which focuses largely on deliberative decision-making with tangible, quite immediate rewarding outcomes, is not well suited to understanding political choice.

In contrast to this focus on deliberative choice about near-term reward arising from rational choice theories in economics, political scientists have emphasized the notion that political decision-making is driven by the use of heuristics or cognitive shortcuts (Popkin, 1991; Sniderman et al., 1993; Lupia, 1994). Politics requires citizens to answer difficult questions involving multiple dimensions and a high degree of uncertainty (Kuklinski and Quirk, 2000). The motivation and cognitive work required may exceed the amount of effort citizens are willing to invest (Fiske and Taylor, 1984). The expected benefit of voting for the best candidate is clearly minimal compared with that of choosing, say, the best car. At the same time, the costs in terms of time and energy are much higher, given the barrage of information that confronts voters during an election campaign. Indeed, Downs (1957) argued that most voters are “rationally ignorant” about politics. Heuristics may thus be a reasonable response to the complexity of these decision problems, replacing the demands of utility maximization with little more than the identification of alternatives and the use of simple rules of thumb to make a choice. For example, citizens may take cues from interveners and agenda-setters, evaluate the incumbent government on the basis of how the economy is doing, rely on a candidate’s party affiliation or ideology, use a candidate’s party affiliation to infer issue positions or simply judge the candidate based on appearance or social background characteristics (Popkin, 1991; Rahn, 1993; Lau and Redlawsk, 2001).

Although economic frameworks encompass choices based on heuristics, with brand loyalty being one example, neuroeconomics research has largely steered away from what political science would term heuristic choices (e.g., Hauser, 2011), instead focusing on paradigms where choices more clearly hinge on deliberations about subjective value. These differences in focus may explain the limited commonalities we identified in the brain correlates of value in economic and political choice. Indeed, recent work on strategy use in economic choice has pointed to engagement of regions outside the commonly identified value-responsive areas when heuristics guide choice (Venkatraman et al., 2009, 2014). Thus, on the one hand, studies of the neural bases of political choice might benefit from testing choices more likely to require deliberation, and on the other, neuroeconomics might benefit from a broader focus, encompassing heuristic choice mechanisms, including considering relevant attributes of individual decision-makers, if the goal is a general model of decision-making in the brain.

A second important distinction between economic and political behavior relates to the characteristics of choice outcomes. In the classical view, economic behavior is about people maximizing value; that is, they make decisions based on the personal payoffs they expect. This view has been complemented and modified over time to include non-monetary factors such as emotion, loyalty, and ambivalence, but it characterizes the essence of economic behavior (Opaluch and Segerson, 1989). Political behavior, on the other hand, involves people’s preferences about aspects of the organization and structure of collective life that may or may not affect them personally. Not only do the consequences of a vote potentially affect everyone in a given society, but the outcome and its expected benefits depend on the decisions of others.

The link between individual choice and outcome is further weakened by the fact that citizens often lack reliable information about the implications of the choices that they are asked to make. For example, in deciding which party to vote for, voters need to compare the expected future performance of the competing parties. However, what parties pledge to do on the campaign trail may not be a reliable guide to what they will actually do in office. Thus, politics provides little in the way of feedback on the correctness of any given choice (Kuklinski and Quirk, 2000), with any feedback distant in time and space from the decision event. Thus, many political decisions may be associated with low confidence in the eventual outcome, a factor that seems to modulate reward-related signals in vmPFC in economic paradigms, for example (Lebreton et al., 2015). Although laboratory economic experiments may have largely hypothetical outcomes (i.e., only one trial might be played “for real”), the limited outcome feedback related to individual political choices seems a potentially important point of distinction with most economic paradigms, in the lab and in life.

It is telling that none of the neuropolitics studies we identified in this systematic review were captured by the recent comprehensive review of fMRI studies of subjective value (Bartra et al., 2013). This is for the simple reason that none used the key word “reward.” Neuroeconomic work has relied heavily on an extensive body of converging research on the brain basis of reward and feedback-driven learning in humans and animal models (Schultz, 2001, 2002; Fiorillo et al., 2003; Cardinal and Howes, 2005; Tom et al., 2007). The conditions under which this can be applied meaningfully, if at all, in neuropolitics research, deserve consideration. One interesting area of potential overlap in this regard might be the case of political donations, as these involve an individual assigning economic (reward) values to political causes.

The consistent engagement of brain reward circuits in neuroeconomic choice likely reflects the fact that such choices are often followed immediately by primary (e.g., food) or secondary (e.g., money) rewards in the experiment, or are cued by stimuli that have such associations in real life. This coincidence of choices and reward is known to drive the release of dopamine in both the basal ganglia and frontal lobes, neural events that underpin value-driven learning (Dagher and Robbins, 2009). On this logic, one could speculate that subjective value correlates of political choices may be more likely to overlap with the signals seen in economic choices when stimuli are used that are more tightly associated with reward or punishment feedback in real life. Qualitative assessment of the few neuropolitics studies that do report foci within the ‘economic’ subjective value-related ROIs may be useful for hypothesis generation in this regard. The two neuropolitics studies that reported activation foci overlapping with positive subjective value in the basal ganglia signals in the reference meta-analysis were by Gozzi et al. (2010) and Tusche et al. (2013). The first selected subjects on the basis of political interest, and asked them to evaluate political statements. The behavioral measure of positive subjective value in this study was the contrast between agreement and disagreement with a statement, in high versus low political interest individuals. This focus on highly motivated subjects may explain why the study found activation overlapping with economic reward-related signals. Tusche et al. (2013) indexed positive subjective value through positive *post-hoc* ratings of well-known politicians whose faces were presented to subjects while they were engaged in a distractor task in the scanner. This design may have focused on ‘automatic’ reward associations similar to the associations triggered by well-learned stimuli in other rewarding contexts. The use of social stimuli (faces) may be particularly effective at eliciting automatic value-related brain activity (Smith et al., 2010) similar to that seen with other cues for primary reward (pictures of food, for example).

The two political studies that reported activation foci for negative subjective value that corresponded to the regions associated with negative subjective value in economic paradigms also used face stimuli. Kaplan et al. (2007) measured negative subjective value by asking participants to look at photographs of politicians from an opposing party. Spezio et al. (2008) asked subjects to vote for unfamiliar politicians based on photographs of their faces, and reported activations related to the candidate that the subject did not vote for in a mock election. In both cases, activity in the insula was correlated with viewing the photograph of a disliked politician (as measured by party preference or vote, depending on the study). Given the putative role of the insula in emotional processing and disgust in particular (Phan et al., 2002; Wright et al., 2004), these results may reflect a ‘distaste’ response either to the specific (known) candidate, or more generally to social characteristics expressed by faces (in the case of unknown candidates).

Overall, this systematic review highlights both points of contact and points of departure between existing research on decision-making grounded in studies of economic value and reward and the emerging literature examining the brain bases of political behavior. On the one hand, identifying the conditions under which brain circuits associated with value defined in economic choice are also engaged in political choice will be informative for neuropolitics and decision neuroscience more generally. On the other, the field of neuroeconomics would benefit from broadening its scope to address aspects of decision-making that have not been a major emphasis to date, but are clearly important in political behavior, such as heuristics and how person-specific characteristics influence decision-making. Finally, given the complexity of political behavior, we can expect that understanding its brain basis will need more than insights from decision neuroscience. Social neuroscience in particular has obvious relevance to many aspects of politics considered beyond the narrow perspective of choice that was the organizing principle of this systematic review.

## Author Contributions

SK carried out the literature review and data synthesis, and drafted the manuscript. DM, DS and EG contributed to the conception of the review, interpretation of the findings, and writing of the manuscript. JM and JK contributed to the quantitative meta-analysis and revised the manuscript. LF conceived of the review, coordinated interactions with co-authors, contributed to synthesis of the findings, and revised the manuscript.

## Conflict of Interest Statement

The authors declare that the research was conducted in the absence of any commercial or financial relationships that could be construed as a potential conflict of interest.
